# Postgraduate corner: Continuing medical education

**DOI:** 10.4103/0019-5545.44755

**Published:** 2008

**Authors:** Chittaranjan Andrade

**Affiliations:** Department of Psychopharmacology, National Institute of Mental Health and Neurosciences, Bangalore - 560 029, India

## CME QUESTIONS

- The premenstrual syndrome describes the cognitive, emotional, and physical symptoms that women commonly experience shortly before the onset of menstruation. The disorder has been known by different names, including late luteal phase dysphoric disorder (LLPDD) and premenstrual dysphoric disorder (PMD or PMDD). Many treatments have been described; these include antidepressant drugs, diuretics, oral contraceptives, calcium, vitamin supplements, exercise, and others. With this background, mark *True* or *False* against each of the following statements:
  1. SSRIs are no better than placebo for PMD.
  2. Antidepressant medication is effective in PMD even if administered only during the luteal phase of the menstrual cycle.
- Topiramate is one of the most recent treatments to be introduced for the management of alcohol dependence. How does it act? Alcohol increases inhibitory GABAergic neurotransmission and decreases excitatory glutamatergic neurotransmission; dopamine, at least in part, mediates the rewarding effects associated with alcohol intake. Topiramate exerts similar GABAergic and glutamatergic effects. It increases GABAergic neurotransmission by action at a nonbenzodiazepine site on the GABA-A receptor and decreases glutamatergic activity by blocking AMPA and kainate receptors. However, unlike alcohol, topiramate indirectly inhibits the release of dopamine in the mesocorticolimbic system. Thus, topiramate can substitute for the GABAergic and glutamatergic effects of alcohol, thereby blunting the neuronal hyperexcitability associated with alcohol discontinuation. And, topiramate can block alcohol-mediated dopamine release, thereby decreasing the rewarding effects of the beverage. Both these actions can be therapeutic in alcohol-dependent patients. With this background, mark *True* or *False* against each of the following statements:
  1. Topiramate therapy is preferably instituted after withdrawal from alcohol.
  2. In alcohol-dependent patients, the target dose of topiramate is 100 mg/day.
  3. From a medical and psychological perspective, controlled drinking could be an acceptable goal of topiramate therapy.
  4. Smoking behavior may decrease in alcohol-dependent patients who receive topiramate.
  5. The efficacy of topiramate remains to be proven in alcohol-dependent patients in conventional clinical practice.

## CME ANSWERS

### A) Treatment of premenstrual dysphoric disorder

Answers: 1. False; 2. True.

#### 1. Efficacy of SSRIs in PMD

SSRIs are well-established, FDA-approved treatments for PMD. Shah *et al*.[1] described a systematic review and meta-analysis of SSRI treatment for the premenstrual syndrome. There were 29 studies of 2964 women which were eligible for analysis. SSRIs were found superior to placebo (odds ratio, 0.40; 95% confidence intervals, 0.31–0.51). No SSRI appeared better than another.

#### 2. Luteal phase treatment for PMD

SSRIs have been used in continuous treatment regimens as well as in intermittent dosing schedules. In the former context, they are administered all through the menstrual cycle. In the latter context, they are administered only during the second half of the cycle, that is, during the luteal phase, or even during the late luteal phase, only. In their meta-analysis, Shah *et al*.[1] found that luteal phase SSRI dosing was effective, but less so than continuous dosing.

### B) Topiramate for alcohol dependence

Answers: 1. False; 2. False; 3. True; 4. True; 5. True.

#### 1. Topiramate and alcohol discontinuation

Two large, randomized, double-blind, placebo-controlled studies have demonstrated the efficacy of topiramate in the alcohol-dependence syndrome.[2,3] In neither trial was abstinence from alcohol a prerequisite for the initiation of topiramate therapy. In fact, one of the important advantages of topiramate is that it can be prescribed even to patients who continue to drink.

#### 2. The target dose of topiramate

In both the major topiramate trials,[2,3] the target dose of the drug was 300 mg/day. Having said so, it must be acknowledged that in neither trial did all patients reach the target dose. For example, in the first trial,[2] 16% of patients did not reach 300 mg/day; in the second trial,[3] the average dose attained was only 170 mg/day. The only dose-ranging study on the subject was inconclusive because it was underpowered.[4]

Patients were more likely to attain the target dose in the first trial[2] because the upward dose titration schedule was more gradual, spanning an 8-week period. Patients were less likely to attain the target dose in the second trial,[3] and were more likely to drop out because of adverse events, because the upward dose titration was more rapid, spanning just 6 weeks. Flexible dose studies and dose-ranging studies are now required to identify the ideal dose titration schedule and the ideal target dose.

#### 3. Topiramate and controlled drinking

In the first topiramate trial,[2] relative to patients receiving placebo, patients who received topiramate had 2.9 fewer drinks per day, 3.1 fewer drinks per drinking day, 27.6% fewer heavy drinking days, and 26.2% more abstinent days. All these findings were statistically and clinically significant. Patients receiving topiramate also had significant reductions in craving, drinking obsessions, automaticity of drinking, and interference due to drinking. In the second trial,[3] the percentage of heavy drinking days dropped from 82 to 44% with topiramate, and from 82 to 52% with placebo; depending on the method of analysis, the advantage for topiramate ranged from 8–18%, and became significant between weeks 2 and 4. Thus, rather than promote abstinence, in both trials topiramate appeared to reduce problem drinking.

In both trials, controlled drinking with topiramate was associated with medical and psychological benefits. Data from the first trial showed that topiramate significantly increased well-being, abstinence, and overall life satisfaction; importantly, topiramate also significantly reduced harmful drinking consequences. These benefits developed in parallel to the significant decrease in the percentage of heavy drinking days.[5] Data from the second trial showed that topiramate was associated with better overall clinical health on global assessments, fewer harmful consequences of drinking, greater reduction in obsessional thoughts and compulsive behavior regarding alcohol use, greater psychosocial well-being, better quality-of-life (including in leisure and household activities), greater reduction in liver enzyme and plasma cholesterol levels, significant reduction in systolic blood pressure (by nearly 10 mm Hg), significant reduction in diastolic blood pressure (by nearly 7 mm Hg), and significant reduction in body-mass index (by approximately one unit).[6]

#### 4. Topiramate and smoking

A secondary analysis of data from the first topiramate trial reported that there were 94 patients who smoked at least one cigarette a day.[7] Of these, 45 patients had received topiramate (mean dose, 280 mg/day at the study endpoint) and 49 had received placebo. No guidance for quitting smoking had been provided, nor had a quit smoking date been fixed. Nevertheless, self-reported smoking cessation rates in the topiramate versus placebo groups were 19.4 vs. 6.9% and 16.7% vs. 6.9% at weeks 9 and 12, respectively. The findings were similar when serum nicotine levels were examined as a confirmatory measure. On various parameters, in the topiramate group lesser drinking was significantly associated with lesser smoking. In the placebo group, in contrast, lesser drinking was significantly associated with greater smoking. This finding indirectly implies that patients compensate for drinking less by smoking more, but not if they are receiving topiramate. In other words, topiramate may have a specific inhibitory effect on smoking.[7]

These results must be viewed with caution because of an overinclusive definition of smoking, and because randomization to topiramate versus placebo had not been stratified for smoking status. There is also no assurance that the results can be applied to smokers who do not have a drinking problem. For example, a laboratory study of smokers found that a single 50 mg dose of topiramate actually enhanced subjective experience of reward in overnight-abstinent smokers who received parenteral nicotine.[8] In another laboratory study, also conducted on smokers, topiramate (75 mg/day) was associated with higher subjective ratings of withdrawal after three hours of smoking abstinence; with increased craving after cigarette cue exposure that was no different from that with placebo; and with an enhanced perception of reward (relative to placebo) after a smoked cigarette.[9]

#### 5. Topiramate in conventional clinical practice

In both of the major topiramate trials, patients were volunteers who were recruited through mass media advertisements, in other words, they were preselected for motivation to stop drinking. Furthermore, these patients had no major medical or psychiatric comorbidities and did not use or abuse any substance other than caffeine or nicotine. Thus, they were not representative of patients seen in conventional clinical practice. We do not currently know how effective topiramate is in real-life situations, that is, in patients who may not be well-motivated, and in those with substance abuse and other comorbidities. Available studies of topiramate in clinical practice suggest efficacy but have methodological limitations such as an uncontrolled or nonblind design.[10,11]

## References

[1] Shah NR, Jones JB, Aperi J, Shemtov R, Karne A, Borenstein J (2008). Selective serotonin reuptake inhibitors for premenstrual syndrome and premenstrual dysphoric disorder: a meta-analysis. Obstet Gynecol.

[2] Johnson BA, Ait-Daoud N, Bowden CL, DiClemente CC, Roache JD, Lawson K (2003). Oral topiramate for treatment of alcohol dependence: a randomised controlled trial. Lancet.

[3] Johnson BA, Rosenthal N, Capece JA, Wiegand F, Mao L, Beyers K (2007). Topiramate for treating alcohol dependence: a randomized controlled trial. JAMA.

[4] Miranda R, MacKillop J, Monti PM, Rohsenow DJ, Tidey J, Gwaltney C (2008). Effects of topiramate on urge to drink and the subjective effects of alcohol: a preliminary laboratory study. Alcohol Clin Exp Res.

[5] Johnson BA, Ait-Daoud N, Akhtar FZ, Ma JZ (2004). Oral topiramate reduces the consequences of drinking and improves the quality of life of alcohol-dependent individuals: a randomized controlled trial. Arch Gen Psychiatry.

[6] Johnson BA, Rosenthal N, Capece JA, Wiegand F, Mao L, Beyers K (2008). Improvement of physical health and quality of life of alcohol-dependent individuals with topiramate treatment: US multisite randomized controlled trial. Arch Intern Med.

[7] Johnson BA, Ait-Daoud N, Akhtar FZ, Javors MA (2005). Use of oral topiramate to promote smoking abstinence among alcohol-dependent smokers: a randomized controlled trial. Arch Intern Med.

[8] Sofuoglu M, Poling J, Mouratidis M, Kosten T (2006). Effects of topiramate in combination with intravenous nicotine in overnight abstinent smokers. Psychopharmacology (Berl).

[9] Reid MS, Palamar J, Raghavan S, Flammino F (2007). Effects of topiramate on cue-induced cigarette craving and the response to a smoked cigarette in briefly abstinent smokers. Psychopharmacology (Berl).

[10] Fernandez Miranda JJ, Marina Gonzalez PA, Montes Pirez M, Dmaz Gonzalez T, Gutierrez Cienfuegos E, Antuqa Dmaz MJ (2007). Topiramate as add-on therapy in non-respondent alcohol dependant patients: a 12 month follow-up study. Actas Esp Psiquiatr.

[11] De Sousa AA, De Sousa J, Kapoor H (2008). An open randomized trial comparing disulfiram and topiramate in the treatment of alcohol dependence. J Subst Abuse Treat.

